# Perspectives in the Application of High, Medium, and Low Molecular Weight Oat *β*-d-Glucans in Dietary Nutrition and Food Technology—A Short Overview

**DOI:** 10.3390/foods12061121

**Published:** 2023-03-07

**Authors:** Leonid Sushytskyi, Andriy Synytsya, Jana Čopíková, Pavol Lukáč, Lenka Rajsiglová, Paolo Tenti, Luca E. Vannucci

**Affiliations:** 1 Department of Carbohydrates and Cereals, University of Chemistry and Technology, Technická 5, 166 28 Prague, Czech Republic; 2 Institute of Microbiology of the Czech Academy of Sciences, Vídeňská 1083, 142 20 Prague, Czech Republic; 3 Faculty of Science, Charles University, Albertov 6, 128 00 Prague, Czech Republic

**Keywords:** cereal *β*-d-glucans, oats, molecular weight, glycaemia, immunity, microbiome, cancer

## Abstract

For centuries human civilization has cultivated oats, and now they are consumed in various forms of food, from instant breakfasts to beverages. They are a nutrient-rich food containing linear mixed-linkage (1 → 3) (1 → 4)-*β*-d-glucans, which are relatively well soluble in water and responsible for various biological effects: the regulation of the blood cholesterol level, as well as being anti-inflammatory, prebiotic, antioxidant, and tumor-preventing. Numerous studies, especially in the last two decades, highlight the differences in the biological properties of the oat *β*-d-glucan fractions of low, medium, and high molecular weight. These fractions differ in their features due to variations in bioavailability related to the rheological properties of these polysaccharides, and their association with food matrices, purity, and mode of preparation or modification. There is strong evidence that, under different conditions, the molecular weight may determine the potency of oat-extracted *β*-d-glucans. In this review, we intend to give a concise overview of the properties and studies of the biological activities of oat *β*-d-glucan preparations depending on their molecular weight and how they represent a prospective ingredient of functional food with the potential to prevent or modulate various pathological conditions.

## 1. Introduction

**Figure 1:**
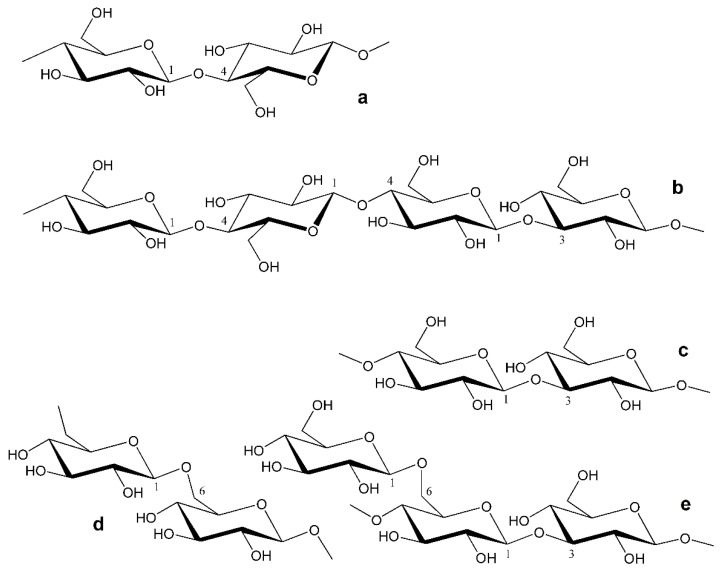
Structure of common *β*-d-glucans: (**a**) cellulose, (**b**) cereal mixed-linkage (1 → 3) (1 → 4)-*β*-d-glucan, (**c**) microbial (1 → 3)-*β*-d-glucan, (**d**) (1 → 3)-*β*-d-glucan from lichens, (**e**) branched fungal or seaweed (1 → 3) (1 → 6)-*β*-d-glucan.

*β*-d-Glucans are a group of structurally diverse carbohydrate polymers composed of *β*-d-glucopyranosyl residues. These polysaccharides are extracted at high yields from fungal biomass, i.e., higher fungi, molds, and yeasts [1,2,3], as well as from higher plants [4], lichens, and algae [5,6]. These polysaccharides are usually structural components of the cell wall or serve as energy stores. Furthermore, various microorganisms are producers of *β*-d-glucans, which they release into the environment as exopolysaccharides. *β*-d-Glucans differ in the configuration of glycosidic bonds and branches depending on origin [1,2,3,4,7]. To date, there are five common structures of natural *β*-d-glucans:

- cellulose or (1 → 4)-*β*-d-glucan [8,9,10], the most abundant polysaccharide in nature, produced by higher plants, marine algae, some invertebrates, and microorganisms (Figure 1a);
- non-branched mixed-linkage (1 → 3) (1 → 4)-*β*-d-glucans [7,11,12,13] from lichens and higher plants, in which both types of linkages are nonrandomly distributed along the chain (Figure 1b);
- (1 → 3)-*β*-d-glucans [14,15,16] produced by fungi and microorganisms (Figure 1c);
- (1 → 6)-*β*-d-glucans [17,18] from lichens and some fungi (Figure 1d);
- branched (1 → 3) (1 → 6)- *β*-d-glucans of various complexity (in terms of branching) from fungi [19,20,21,22,23,24,25,26,27,28,29] and algae [5,6], usually consisting of 1,3-linked *β*-d-glucopyranosyl units in the backbone and terminal *β*-d-glucopyranosyls as side chains linked at the *O*-6 position (Figure 1e).

Branched structures are typical for higher fungi, molds, yeasts, and brown algae [1,6], while mixed-linkage structures are found in plants, especially cereals [4] and lichens [7]. The fungal branched *β*-d-glucans commonly have a higher degree of structural variability, mainly determined by the configuration of the glycosidic bonds in the backbone, the degree of branching, and the size and structure of the side chains [1]. Despite similarities in configuration, laminarins, the storage *β*-d-glucans from brown algae, differ from the *β*-d-glucans of fungal cell walls by a much lower *M*_w_ and by the presence of terminal d-mannitol units in some backbone chains [5]. Animals do not produce any types of *β*-d-glucan except tunicates, a group of marine animals capable of producing cellulose [10].

Generally, as reported, mushroom- and cereal-based foods containing these polysaccharides are beneficial for health, with anticarcinogenic, antiviral, and immunostimulatory properties. The names of some well-studied bioactive *β*-d-glucans correspond to their origin. Good examples are the trade names for the branched *β*-d-glucans and their protein complexes obtained from various fungi, such as krestin from *Coriolus versicolor* [23], lentinan from *Lentinus edodes* [24], schizophyllan from *Schizophyllum commune* [27], pleuran from *Pleurotus ostreatus* [26], grifolan from *Grifola frondosa* [22], and many others (Table 1). These products are sold as dietary supplements with immunostimulant, antioxidant, antidiabetic, and anticarcinogenic effects and have long been used by traditional medicine, especially in Asia [30]. In addition to the yeasts and seaweeds mentioned earlier, other natural sources of commercial *β*-d-glucans include lichens, microalgae, and bacteria, in addition to the yeasts and seaweeds mentioned earlier. Regarding the mixed-linkage (1 → 3) (1 → 4)-*β*-d-glucans from commercially grown cereals, they are sold mainly in the form of fortified flours or concentrates [31,32,33].

Considering the prices of many products, including *β*-d-glucans, one should remember that oats belong to the cheapest grains available in the Western world, and people can meet their daily *β*-d-glucan needs with affordable oat flakes or porridges.

As we will further discuss, the processing of oats has a crucial impact on the main parameters of *β*-d-glucan, such as *M*_w_, viscosity and availability to immune cells or other biological or biomolecular targets.

## 2. Oats: Health Benefits and the Source of Mixed-Linkage *β*-d-Glucan

The oat (*Avena sativa*) is a well-known cereal and one of the first cultivated plants on Earth. Humans used this crop extensively in the last century, but in the present time, the area under oats in some countries has drastically decreased. Currently, world oat production is about 22 million tons per year (compared to 46.9 million tons in 1961 [34]), less than barley at 170 million tons per year and much less than the 772 million tons of wheat in 2021. The most widely grown species are common oat *Avena sativa* L., naked oat *Avena nuda* L., and red oat *Avena byzantine* [35,36].

Oat groats are rich in protein (usually 13–20%) and a source of unsaturated fatty acids. These properties make it one of the most popular and healthy cereals as plain oat porridge or in a mixture for quick-prepared mueslis. In the EU, Canada, and the U.S., oats are also found among gluten-free commodities, supplying the production line for gluten-free oat-containing foods, and studies show the general safety of this cereal for celiac disease [37]. The human consumption of oats supports the growth of beneficial gut microbiota such as bacteria of the genera *Bacteroides* and *Prevotella*. In addition, a diet containing oats may increase levels of butyric acid and propionic acid, which can positively impact the gut microbiota as well as diabetes mellitus, the latter by altering glucose absorption [38]. In this context, oat fibers can improve brain function by stimulating the microbiota to produce more short-chain fatty acids (SCFA), which can cross the blood–brain barrier with neuroprotective effects [39]. Gao et al. [40] found that feeding oat fiber to male mice with LDL receptor knockout (LDLR−/−) for 14 weeks significantly improved the cognitive behavior of animals. Using an atherosclerosis model, they found that a high fat, high cholesterol diet promoted neuroinflammation in LDLR−/− mice, whereas 0.8% dietary oat fiber had an anti-inflammatory effect. This phenomenon correlated with the inhibition of GFAP and IBα1 expression in the cortex and GFAP decrease in the hippocampus. Both proteins are involved in Aβ plaques associated with Alzheimer’s disease and cerebral amyloid angiopathy. In this study, Aβ plaques developed in the LDLR−/− mice regardless of oat fiber consumption; however, oat fiber administration decreased the Aβ plaque formation.

Oats also contain natural antioxidants such as tocopherols, tocotrienols, sterols, and phenolic acids. In addition, alkaloids, such as avenanthramides, also contribute to the antioxidant activity of oat-containing food [41].

Similarly to other cereals, viscous mixed-linkage (1 → 3) (1 → 4)-*β*-d-glucans are also present in oats [42]. The *β*-d-glucans from oats, barley, rye, and wheat have nearly identical structural properties, as proved by NMR correlation spectroscopy and methylation analysis. However, these polysaccharides exhibit differences in linkage distribution reflected in the ratio of cellotriosyl to cellotetraosyl blocks, i.e., ~1.5–2.3 in oats, ~2.7–3.9 in barley and rye, and ~3.8–4.8 in wheat [43]. Consequently, this ratio is a fingerprint of the structure of cereal *β*-d-glucans [44].

The possible correlation between the *M*_w_ of these polysaccharides and their beneficial effects appears of particular interest in the *β*-d-glucan from oats, especially if looking for their impact on digestion and immune response [45,46,47,48]. The increase in studies comparing the properties of *β*-d-glucans with different *M*_w_ but from the same source makes oats a perfect subject for investigations. They are of high availability, low cost, and ease of *M*_w_ modification.

## 3. Physical Properties and *M*_w_ of *β*-d-Glucans

The solubility of *β*-d-glucans in aqueous and nonaqueous media depends on their configuration, degree of branching, and *M*_w_. In aqueous solutions, *β*-d-glucan macromolecules can adopt different conformations: random coil, helical (single, double, and triple helix), aggregated, and rod-shaped [49,50]. Oat *β*-d-glucans are suitable for various forms of food due to high water solubility of about 70% (*w/w*) and even 80% (*w/w*) [51], while barley *β*-d-glucans, for example, are soluble at 15-20% (*w/w*) [52]. Other sources report 65% (*w/w*) and 82% (*w/w*) of soluble *β*-d-glucans for barley and oats, respectively [53,54].

As with some other compounds found in the cell wall, *β*-d-glucan macromolecules can be obtained from plant or fungal biomass by extraction with aqueous media, and the *M*_w_ of these polysaccharides depends on the origin and the method and conditions of extraction. Cell walls decompose during the extraction process, and their macromolecular components, including *β*-d-glucans, are released depending on their solubility in the extraction medium and hence on their structure and *M*_w_. Conventionally, the *β*-d-glucan polymers with *M*_w_ up to 500 kDa are indicated as Low Molecular Weight (LMW), while the 1000 kDa or more as High Molecular Weight (HMW) polymers. In some articles, *β*-d-glucans with intermediate (medium) *M*_w_ in a range between 500 and 1000 kDa and even in a broader range are defined as Medium Molecular Weight (MMW), but this is rarer in comparison to the most commonly used definitions of LMW and HMW *β*-d-glucans [55].

*β*-d-Glucans from oats and barley can self-aggregate into microgels suitable for interaction with other biomolecules, such as proteins [56]. It seems that *β*-d-glucans lose their viscosity upon contact with the gut microflora, probably due to a decrease in their *M*_w_ [57], and the consumption of *β*-d-glucan-enriched foods always leads to the appearance of *β*-d-glucan fractions with different *M*_w_. Rebello et al. [58] confirmed the benefits of instant oatmeal compared to ready-to-eat oat-based breakfast cereals, as oatmeal contains a higher proportion of HMW *β*-d-glucans (*M*_w_ ~381–398 kDa), whereas ready-to-eat oat cereals more likely contain *β*-d-glucans with *M*_w_ = 217–225 kDa. In oatmeal, the hydrated molecules form larger spheres, which explain the increased viscosity of the food. For the normalization of digestion, oat HMW *β*-d-glucans are beneficial due to their rheological properties. They increase the viscosity of the chyme in the intestine and provide more favorable structural properties for bakery products, i.e., optimal firmness and texture [59]. Brummer et al. [60] studied gels and viscous solutions of mixtures of HMW/LMW oat *β*-d-glucans at different concentrations (3, 4, and 5%) and ratios (0:1, 1:3, 1:1, 3:1, and 1:0). The 1:1 mixture showed the highest storage moduli (*G*′), whereas for the pure HMW *β*-d-glucan the *G*′ was minimal. The peak melting point was highest for pure LMW *β*-d-glucan and decreased with increasing amounts of HMW *β*-d-glucan. The strongest gels formed at all concentrations of the mixtures with ratios 1:3 and 1:1 HMW to LMW *β*-d-glucans. The gel prepared from pure LMW *β*-d-glucan showed an ordered microstructure, which is not pronounced in the HMW/LMW *β*-d-glucan mixtures. Gels with added glucose are denser and firmer than those without glucose. Different ratios of HMW/LMW *β*-d-glucans in foods can thus tailor the rheological properties of products to consumer needs.

The provided results confirmed that oat *β*-d-glucans could serve in food technology as a gelling agent, emulsifier, and texturizer. Depending on *M*_w_, this polysaccharide, which has good solubility in water, can be used in cosmetics and medicine more broadly than other cereal *β*-d-glucans since it is less contaminated with gluten.

## 4. Possible Molecular Mechanisms of Oat *β*-d-Glucan Activities

The *β*-d-glucans are known for their immunomodulatory properties [61], but their biological effects can vary depending on the source, chemical complexity, and, possibly, bioavailability [1,3,62]. Although mushrooms and cereals are two principal sources of *β*-d-glucans, the former exhibit much more complex, often branched structures of these polysaccharides that may enhance their immune activation properties and explain why mushroom *β*-d-glucans were considered potent anti-cancer agents [1,63]. Nevertheless, *β*-d-glucans from all sources can trigger the innate immune system due to their ability to activate professional phagocytes, primarily macrophages [64]. The effects of fungal (1 → 3) (1 → 6)-*β*-d-glucans on the immune system have long been known [65]. For cereal (1 → 3) (1 → 4)-*β*-d-glucans, these properties are less understood [42,62,66,67], although in vitro tests have shown that cereal *β*-d-glucans can elicit cytokine secretion, phagocytosis, the cytotoxicity of isolated immune cells, and the activation of the complement system [51,68]. The protective effect of these polysaccharides against intestinal parasites and bacterial and viral infections is evident [69,70], as well as their synergistic effect in antibody-dependent cellular cytotoxicity [71]. The modulation of cytokine responses depends on the block sequence of glycosidic bonds of cereal (1 → 3) (1 → 4)-*β*-d-glucans expressed by the ratio of cellotriosyl and cellotetraosyl units in the chain [68]. This ratio affects the solubility and tendency of this polysaccharide to form aggregates in aqueous systems. The low solubility and high ability to aggregate lead to an enhanced immune response.

As with cellulose, cereal mixed-linkage *β*-d-glucans are not digestible in the human gastrointestinal tract but can act as the carbon source for some species of bacteria in the human gut and are effectively reduced by them. Raw cellulose does not uptake water and does not gel. Consequently, it usually does not participate in digestion but only supports the formation of feces. In contrast, (1 → 3) (1 → 4)-*β*-d-glucans, either in their long or short fragments, are transferred by enterocytes to the lymph, where macrophages subsequently engulf them activating a process of degradation lasting more than four days [72]. After the digestion of cereal foods, residues of these polysaccharides appear in the serum.

In the intestinal lumen, pattern recognition receptors (PRRs) recognize *β*-d-glucans coming with food [73]. The primary PRR for the water-insoluble fraction of *β*-d-glucans is Dectin-1 expressed on the surface of macrophages and dendritic cells (DC), while the water-soluble portion of *β*-d-glucans can bind to Complement Receptor 3 (CR3) [13]. Pattern recognition receptors can recognize a variety of evolutionary conservative molecules assigned as pathogen-associated molecular patterns (PAMPs), and since *β*-d-glucans generally fall into this category, it is their primary mechanism of immunostimulatory effect [64]. The link to these receptors activates phagocytosis. In addition, it activates downstream signal pathways: Syk-CARD9, NF-kB, Raf1 by Dectin-1, MAPKs, NF-kB, PI3K-Akt, p38 by CR3, and MYD88-NF-kB/MAPK by TLR-4 and Dectin-1 [74,75]. After the pre-digestion of oat *β*-d-glucan with enzyme endo-1,4-*β*-glucanase (EC 3.2.1.4), the cleavage of the internal *β*-(1 → 4) linkages follows. Interestingly, this process leads to a more intense activation of Dectin-1 receptors due to a higher abundance of *β*-(1 → 3) bonds becoming accessible for this receptor. Moreover, the reduced *M*_w_ and particle size of the pre-digested *β*-d-glucan facilitates its availability to even more receptors. This effect is especially pronounced in the DC [76].

Choromanska et al. [66] described the relevant cytotoxic effect of oat *β*-d-glucan on cancer cells. Melanoma cells Me45 can internalize HMW oat *β*-d-glucan (*M*_w_ 1338 kDa) with the assistance of GLUT1 (Glucose Transporter Type 1) protein. Melanoma cells demonstrate the increased expression of GLUT1, and glucose-based polymers can be absorbed more intensively compared to normal cells. Electroporation enhanced the cytotoxic effect of *β*-d-glucan by reducing glucose uptake in media with WZB117, a GLUT1 inhibitor. Cell viability was high with simultaneous incubation with oat *β*-d-glucan and WZB117 but without electroporation. It is not clear if LMW *β*-d-glucan would show a better or comparable effect, but potentially as it is more water soluble it could show better results. Despite this, it is clear that there are multiple ways in which *β*-d-glucans can act on normal and cancer cells.

It is important to note that direct comparisons of cereal and fungal *β*-d-glucan activities are rare. Several groups provided many results reflecting the importance of designing a preparation of *β*-d-glucans with various ranges of *M*_w_, suitable for improving different conditions or as immunostimulants, as an alternative to current types of *β*-d-glucan fibers sold as food supplements or advertised as multi-purpose remedies.

## 5. Preparation of Oat *β*-d-Glucans with Various *M*_w_

Cereal (1 → 3) (1 → 4)-*β*-d-glucans with different *M*_w_ are common in bread, flakes, and beverages [4], and the range of *M*_w_ is highly dependent on processing technologies [77,78], including the temperature conditions [79,80,81], enzymatic treatment, fermentation, storage [82], and also on the oat breed [83]. In addition, the influence of environmental factors is also hard to estimate [36,84].

Amylolytic and proteolytic enzymes can improve the final viscosity of *β*-d-glucan extracts from oat-containing products [72]. For HMW *β*-d-glucan, viscosity increases by facilitating the release of this polysaccharide from the food matrix. On the contrary, for partially degraded LMW *β*-d-glucan, the final viscosity decreased without the contribution of starch and proteins. Microbial α-amylase, i.e., endo-α-1,4-glucanase (EC 3.2.1.1), produced by *Bacillus licheniformis* and pancreatin (EC 3.4.21.70) from pork pancreas, was the most effective for the release of oat *β*-d-glucan from food matrices (Table 2). Thus, these enzymes can be used in in vitro models to estimate the viscosity of oat-containing products in the lumen of the upper intestine.

Processing with endo-*β*-glucanases of various origins, which catalyze the cleavage of glycosidic linkages between *β*-d-glycopyranosyl residues, leads to the degradation of *β*-d-glucan macromolecules [72,85,86,87,88,89,90]. Four classes of these enzymes differ in the type of cleavable glycosidic bond in the polysaccharide chain [91,92]:

- endo-*β*-1,3-1,4-glucanases or lichenases (EC 3.2.1.73);
- endo-*β*-1,3(4)-glucanase (EC 3.2.1.6);
- endo-*β*-1,3-glucanases or laminarinases (EC 3.2.1.39);
- endo-*β*-1,4-glucanases or cellulases (EC 3.2.1.4).

Besides laminarinases, these enzymes can degrade HMW oat *β*-d-glucan (Table 2) up to MMW/LMW oat *β*-d-glucans or glucooligosaccharides [91,92]. Adding lichenase (EC 3.2.1.73) to the mixture of hydrolytic enzymes used for releasing *β*-d-glucan from oat matrices drastically reduced the viscosity of the slurry because of the degradation of this polysaccharide [89]. Oat bran processing with a mixture of *β*-glucanases from *Aspergillus sp.* and *Trichoderma longibrachiatum* led to obtaining *β*-d-Glucans of different *M*_w_ [90]. Whole wheat flour used in bread baking contains active endo-1,3(4)-*β*-d-glucanase (EC 3.2.1.6) that can degrade *β*-d-glucans to 10% of their initial *M*_w_ in just 30 min of incubation under 50 °C [86]. Such processes as steaming and heating could contribute to the deactivation of this enzyme and thus can increase the *M*_w_ of *β*-d-glucan extracted from oats [87]. High pH can inactivate *β*-glucanase and thus increase the *M*_w_ of extracted *β*-d-glucan and the solubility of other oat flour components. By contrast, low pH values can cause the hydrolysis of glycosidic bonds and reduce the *M*_w_ of extracted polysaccharides. Extraction yield can also increase in such a way due to the higher solubility of LMW *β*-d-glucan [93]. The freeze-milling of oat bran is also effective for obtaining LMW *β*-d-glucan from oats without pH alteration or enzymes [94].

After extraction, the physical properties and *M*_w_ of isolated *β*-d-glucan undergo modifications by various treatments [50]. For example, the enzymatic treatment and micro-fluidization of oat *β*-d-glucan reduces both *M*_w_ and the average size of particles, increases extractability up to 80%, water retention capacity, decreases the viscosity of the *β*-d-glucan suspension, and increases colloidal stability [95]. Roubroeks et al. [88] used cellulase (EC 3.2.1.4) from *Trichoderma* sp to partially depolymerize oat *β*-d-glucan. The enzyme-treated polysaccharides had *M*_w_ ranging from 2.2 to 213.9 kDa and intrinsic viscosity from 7 to 316 mL/g, and previously semi-flexible chains adopted extended conformation. Bae et al. [89] prepared oat *β*-d-glucan hydrolysates (*M*_w_ 370–1450 kDa) using cellulase treatment affecting swelling and affinity for fat and bile acids.

Shah et al. [96] and Hussain et al. [97] used γ-radiation to modify *β*-d-glucan (*M*_w_ ~200 kDa) previously extracted from oats and obtained fractions of various *M*_w_. The γ-ray irradiation of this polysaccharide at doses 3, 6, 9, 12, and 15 kGy yielded 144, 108, 80, 61, and 52 kDa fractions, respectively [97]. With an increased γ-radiation dose, the fractions acquired increased antioxidant activity and had a cytotoxic effect against cancer cell lines, particularly Colo-205 and MCF7 [96,97]. All these fractions were found safe for normal cells and presented improved solubility.

Sun et al. [98] studied three oat LMW *β*-d-glucan preparations (*M*_w_ 1.83, 4.55, and 27.5 kDa) obtained by acid degradation of the initial HMW polysaccharide. The structure of oat *β*-d-glucan did not alternate by acid degradation, but the resulting change in *M*_w_ influenced the viscosity of polysaccharide solutions.

It is worth mentioning that a broad range of products containing oats already exists, relying on well-established technological processes. Nonetheless, the *M*_w_ of *β*-d-glucan in products that have been on the market for decades is either never mentioned or can only be predicted or judged by viscosity. In addition to developing new technologies for tailoring the *M*_w_ of oat *β*-d-glucan in future products, it is necessary to study the existing ones to obtain an idea about the possible additional benefits of each product on the market.

**Table 2:** Overview of enzymes used in treatment with oat-containing products and oat *β*-d-glucans.

Enzyme	EC Number	Source	Cleavage of Bonds	Effect on Oat *β*-d-Glucans	References
Pancreatin	EC 3.4.21.70	Pork pancreas	Internal peptide bonds	Release of *β*-d-glucan from oat products (the upper intestine model)	[72]
Endo-1,4-α- glucanase (α-amylase)	EC 3.2.1.1	*Bacillus licheniformis*	Internal 1 → 4 bonds		
Endo-1,4-*β*- glucanase (cellulase)	EC 3.2.1.4	*Aspergillus niger* *Trichoderma* sp.	Internal 1 → 4 bonds	More potent activation of Dectin-1	[88,89,90]
				Decrease in *M*_w_ Reduction of viscosity	
Endo-1,3(4)-*β*- glucanase	EC 3.2.1.6	Wheat, rye, barley	Internal 1 → 3- or 1 → 4 bonds		[85,86,87]
Endo-1,3:1,4-*β*- glucanase (lichenase)	EC 3.2.1.73	*Bacillus subtilis*	Internal 1 → 4 bonds		[72]

## 6. Functionalized Oat *β*-d-Glucans

Besides the mineral or enzymatic degradations mentioned above, other chemical modifications can improve the physical properties and biological effects of oat *β*-d-glucan. New functional groups or small molecules are usually attached to polysaccharide chains via primary or secondary hydroxylic groups [50]. In addition, redox reactions can modify oat *β*-d-glucan as well. Chemically modified oat *β*-d-glucans are summarized in Table 3.

De Sousa et al. [82] described the *O*-acetylated derivatives of oat *β*-d-glucan. The acetylation of this polysaccharide increases its swelling and bile acid binding capacities but reduces its viscosity in solutions, gel stickiness, and gel hardness. Acetylated oat *β*-d-glucans with a degree of substitution (DS) of 0.03 to 0.12 are suitable for food applications. In another study, oat *β*-d-glucan was subjected to sequential oxidation and the reductive amination of primary hydroxyl groups to obtain a cationic derivative (DS 0.48) [99]. The modification resulted in an increase in the binding capacity of bile acids compared to the native polysaccharide. In addition, the derivative showed a pronounced antimicrobial effect against *Escherichia coli* and *Bacillus subtilis* and the concentration-dependent inhibition of the angiotensin-converting enzyme (ACE). It also stimulated nitric oxide synthesis in bronchoalveolar lavage experiments. The improved functionality of the derivative is due to its polycationic nature.

Song et al. [100] prepared carboxymethylated (CM) derivatives (DS to 0.06) of oat HMW *β*-d-glucan (*M*_w_ 1300 kDa) by treating native polysaccharide with alkaline ethanolic sodium chloroacetate. This modified polysaccharide effectively improved the tear and tensile strength of the paper, as well as the paper’s folding strength. This approach allowed for the improvement of the physical properties of paper hand sheets: improved tensile index by 51.4%, burst index by 60.7%, and folding endurance by 185.1% after 45 min of carboxymetylation of *β*-d-glucan and 1.5% of CM-glucan in paper compared to a control with non-CM-glucan.

Chang et al. [101] prepared sulfated oat *β*-d-glucan (DS 0.68, *M*_w_ 68 kDa). Compared to the native polysaccharide, the *M*_w_ and viscosity of the derivative significantly decreased, but its solubility increased about twice. The sulfation also reduced the bile acid binding capacity of oat *β*-d-glucan due to the new anionic character. The sulfated *β*-d-glucan exhibited a concentration-dependent anticoagulant activity because of sulfate groups.

The combined action of oat *β*-d-glucan with polyphenols may enhance the resulting biological effects [102,103,104,105]. For example, grafting ferulic acid (DS 0.03–0.18) added DS-dependent antioxidant activity to this polysaccharide. With increasing DS, the antioxidant effect was comparable to ferulic acid itself. Conjugates showed higher stability than ferulic acid under gastrointestinal and colonic conditions. The derivatives were also more potent against human colon cancer cell line HCT-1116 than ferulic acid at equal amounts [103]. The conjugates also demonstrated marked in vitro cytotoxic activity against human colorectal cancer cells.

**Table 3:** Chemical modification of oat *β*-d-glucans.

Reaction	Reagent	Product	DS	Properties Tested	Ref.
Oxidation	TEMPO/NaClO/NaClO_2_	6-carboxy-*β*-d-glucan	0.65	bile acid binding	[106]
	NaIO_4_	*β*-d-glucan dialdehyde			
Oxidation Reductive amination	HCOH NH_4_OAc/NaBH_4_CN	cationic *β*-d-glucan	0.48		[99]
				antimicrobial	
				ACE inhibitor	
Etherification	NaCH_3_COCl	CM *β*-d-glucan	0.06	paper improving	[100]
Esterification	Ac_2_O	*O*-acetylated *β*-d-glucan	0.03–0.12	swelling, rheology	[82]
				bile acid binding	
	HCOH, HSO_3_Cl propylene oxide	sulfated *β*-d-glucan	0.68		[101]
				solubility	
				anticoagulant	
	ferulic acid	*O*-feruloylated *β*-d-glucan	0.03–0.18	stability, anticancer	[103]
				antioxidant	
Maillard reaction	amino acids	conjugates		rheology	[107]
	peptides				

Sun et al. [107] prepared oat *β*-d-glucan conjugates using the Maillard reaction. The conjugation of this polysaccharide with amino acids and peptides led to different effects on its rheological properties. The *β*-d-glucan-amino acid conjugates exhibited shear thinning, while the *β*-d-glucan-peptide conjugates were Newtonian fluids. The apparent viscosity of the former conjugates increased compared to unmodified oat *β*-d-glucan, but for the latter conjugates this value decreased sharply when the shear rate $\dot{\gamma }$ exceeded 10 s^−1^. At the angular frequency *ω* of 1 Hz, *β*-d-glucan-amino acid conjugates showed much higher values of *G*′ than those of the native polysaccharide or its peptide conjugates. At all *ω*, the storage modulus *G*′ exceeded the loss modulus *G*″ for both types of conjugates (7% *w/v*), so they showed elastic behavior.

Various experiments with the modification of oat *β*-d-glucan demonstrated that this polysaccharide is suitable for many applications in food technology and other fields. The biological activities of oat *β*-d-glucans in relation to *M*_w_ are summarized in Table S1 (Supplementary materials).

## 7. Influence on Gastrointestinal Diseases and Inflammation

Many studies in the last few decades searched for the possible nutraceutical applications of LMW and HMW oat *β*-d-glucans, most notably as co-treatments for gastrointestinal diseases such as Crohn’s disease, colitis, enteritis, and gastritis, associated with chronic inflammation.

Żyła et al. [108] modeled Crohn’s disease in rats, i.e., 2,4,6-trinitrobenzenosulfonic acid (TNBSA)-induced colitis, and found that the LMW fraction (*M*_w_ < 100 kDa) of oat *β*-d-glucans was more beneficial for animal weight gain and recovery because the macromolecules of this fraction are more accessible to the immune cells, inducing immunomodulation. Their effectiveness in stimulating Dectin-1 receptors expressed on the surface of dendritic cells and antigen-presenting cells is higher due to an increased proportion of 1,3-linkages. By contrast, in the same study, HMW *β*-d-glucan was more effective in reducing the inflammatory infiltration of mucosal and submucosal lymphocytes because of the ability of this fraction to form a protective layer on the mucosal surface against the irritating agent. When added to food, both LMW and HMW fractions of oat *β*-d-glucan significantly increased the percent of B cells compared to control animals with untreated colitis. The same authors [109] performed a similar experiment on Sprague Dawley rats with TNBSA-induced colitis. The experimental and control animals obtained a diet containing 1% LMW or HMW oat *β*-d-glucan; the control group’s diet was free of this polysaccharide. In animals fed with LMW *β*-d-glucans, the gene expression of pro-inflammatory cytokines (IL-1, IL-6, TNF-α) reduced, and their concentration in the colon was lower compared to control. The authors pointed out that this suppression activity could have a positive therapeutic effect in patients with Crohn’s disease who also have mutations in the gene NOD2/CARD15 complex, an intracellular receptor that activates pro-inflammatory cytokine synthesis. When this gene is mutated, the reduction of inflammation cannot be regulated by anti-inflammatory cytokines, thus leading to persistent inflammation in the gut. High Molecular Weight oat *β*-d-glucan improved the overall recovery of tissue at the histological level. According to the authors, a diet containing oat *β*-d-glucan is neutral for the normal intestine.

Kopiasz et al. [110] studied the effect of supplementation with food containing 1% (*w/w*) of either HMW or LMW oat *β*-d-glucan on hematological and biochemical parameters in mice with early stages of TNBSA-induced colitis. Eighteen groups of animals obtained different diets. The experimental group with colitis was fed with TNBSA and its subgroups contained animals fed additionally with HMW, LMW, or without oat *β*-d-glucans for 3, 7, and 21 days. Similar subgroups were in the healthy control group, but 0.9% NaCl was given instead of TNBSA. The authors mentioned that during the first five days of observation, the control group consumed significantly more food than the group induced to colitis. During the same period, the mice with colitis fed with LMW *β*-d-glucan consumed more food than the colitis mice without *β*-d-glucan in their diet. The analysis of alanine aminotransferase (EC 2.6.1.2), aspartate aminotransferase (EC 2.6.1.1), and alkaline phosphatase (EC 3.1.3.1) activities showed no significant difference in all groups, thus reporting the low hepatotoxicity of 1% *β*-d-glucans in food. Mice in both control and experimental groups that consumed *β*-d-glucans showed a higher percentage of T cells in the lymphocyte population, most evident after three days of supplemented diet intake, especially in the colitis group. After three days of feeding, control mice fed with LMW *β*-d-glucans showed the highest percentage of B cells. Platelet count was higher in the untreated colitis group, which did not obtain *β*-d-glucans, but did not differ among the groups at feeding days 7 and 21. Compared to the control, the percentage of NK cells was significantly higher in mice with colitis receiving *β*-d-glucans in the diet, regardless of their molecular weight. In the control group, an increase in NK cells was induced only by the HMW *β*-d-glucan. These authors [111] carried out a similar experiment with TNBSA on rats and measured the expression levels of protein-1 light chain 3, mammalian isoform B (LC3B), and Caspase-3 (the autophagy markers) and TLR and Dectin-1 receptors in colonocytes. Their results showed that colitis decreased the expression of TLR 4 and Dectin-1, but supplementation with *β*-d-glucans increased the expression of Dectin-1 and TLR 5. The intake of HMW and LMW oat *β*-d-glucans positively influenced the state of rats with colitis. The consumption of feed with HMW and LMW *β*-d-glucans by colitis mice committed to a higher expression of microtubule-associated LC3B, compared to other diets and after 21 days of the experiment this was on a similar level to the control group with a usual diet without *β*-d-glucans. Noteworthily, LC3B is a protein that plays one of the central roles in autophagy, and thus oat *β*-d-glucans can enhance this process in patients with Crohn’s disease. Both fractions of *β*-d-glucans significantly reduced the expression of Caspase-3 in the experimental group compared to colitis mice without *β*-d-glucans in the diet and therefore protected the intestinal barrier via reducing the extent of apoptosis. Kopiasz et al. [112] showed that dietary oat *β*-d-glucans, especially those of HMW, can improve colitis by modulating the expression of chemokines, their receptors, and some Crohn’s disease-associated proteins.

Wilczak et al. [113] modeled enteritis in rats by LPS supplementation in a small-scale animal study. They observed changes in IL-2, IL-10, and TNF-α levels in the rat colon. While LPS can reduce colon level of IL-10, HMW oat *β*-d-glucans fed to LPS-induced enteritis mice were able to normalize IL-10 and thus reduce pro-inflammatory cytokines IL-2 and TNF-α. Błaszczyk et al. [114] studied the transcriptomic profile in the peripheral blood of mice with LPS-induced enteritis and fed them with a diet containing either LMW or HMW oat *β*-d-glucans. The intravenous injection of LPS triggered an innate immune response to bacteria threat by activating TLR4 in the cell membrane of many immune cells. Mice obtaining a diet with HMW oat *β*-d-glucan showed a 2.35-fold decrease in the expression of Granzyme C-like protein compared to the LPS-only mice. The authors connected this with the potential anti-inflammatory activity of HMW oat *β*-d-glucans. About a 12.6-fold expression fall was shown for the *Serp2* gene, while it was about 3.4 times upregulated in LPS-only mice. The *Serp2* gene is responsible for encoding stress-associated endoplasmic reticulum protein family member 2, responsible for numerous pathways involved in the initiation step of inflammation, angiogenesis, and other processes. The authors connect the upregulation of *Serp2* with the inhibition of immune response by binding to the IL-1β-converting enzyme with a reduction of the level of the active cytokine. High Molecular Weight oat *β*-d-glucans can prevent the inhibition of the immune response in LPS-challenged mice. Another gene that underwent elevated expression was *Nlrp1*. Its product usually initiates the formation of an inflammasome complex in cells and appears to inhibit gastrointestinal inflammation and tumorigenesis. The authors noted that *Nlrp1* expression was influenced only by HMW but not LMW oat *β*-d-glucans. Low Molecular Weight oat *β*-d-glucans regulate the synthesis of specific proteins, most notable with the increase in IL34 and decreased prostaglandin E receptor 3, both involved in the inflammatory process.

Suchecka et al. [115] studied the effect of LMW and HMW oat *β*-d-glucans on LPS-induced chronic enteritis in rats. In rats, unlike in mice, healthy control animals fed with HMW *β*-d-glucan had a lower percentage of NK lymphocytes than their untreated counterparts, which suggests a possible species-specific response to the supplementation. The percentage values of total lymphocytes, B lymphocytes, Tc, and granulocytes decreased in animals with enteritis regardless of *β*-d-glucan *M*_w_ supplementation. In enteritis rats, monocytes decreased under HMW oat *β*-d-glucan feeding. In rats with sodium deoxycholate-induced gastritis [116], treated with a diet supplemented with LMW (*M*_w_ 70 kDa) and HMW (*M*_w_ 2180 kDa) fractions of oat *β*-d-glucan, a significant decrease in the levels of lipid oxidation occurred. The total antioxidant status increased in stomach tissue, especially for the HMW fraction. According to the authors, the purified fractions of oat *β*-d-glucans could be more beneficial for gastritis treatment than oat bran because the bran cellulose can cause issues in patients with gastric problems.

Gudej et al. [117] studied the effect of the 30-day supplementation of oat *β*-d-glucan on 129 patients with confirmed chronic gastritis using a diet containing LMW (*M*_w_ 70 kDa) and HMW (*M*_w_ 2180 kDa) fractions of pure oat *β*-d-glucan. The daily consumption of 3 g of *β*-d-glucans resulted in molar mass-dependent changes, mainly in immunological parameters and fecal SCFA concentration. High Molecular Weight oat *β*-d-glucans reduced the mucosal damage, and the normalized concentration of SCFA in feces positively influenced peripheral blood glutathione metabolism and antioxidant parameters.

Oat *β*-d-glucan can thus affect sophisticated molecular signaling involved in gut inflammation. Different *M*_w_ fractions can have a similarly positive effect during colitis or induce autophagy during Crohn‘s disease, but this effect may be less consistent when investigated with a more detailed study of blood cell count and blood protein data. Species differences may also influence the response to the used supplementation. Nevertheless, according to the various shreds of evidence, fundamental differences in the anti-inflammatory action mechanism of HMW and LMW *β*-d-glucans can exist.

## 8. Cholesterol Regulation and Bile Acid Binding

A substantial amount of data regarding the influence of oat *β*-d-glucan on blood cholesterol levels and bile acid binding is available. Forty-nine intervention trials between 1997 and 2019 reported total and LDL cholesterol lowering by oats [46]. These effects of oat *β*-d-glucan depend on their availability, *M*_w_, and viscosity. Gamel et al. [72] showed that the reduction of blood LDL cholesterol was more pronounced after consuming a beverage containing oat *β*-d-glucan than after consuming bread and cookies prepared with the same oat fiber. Compared to food with phytosterols only, oat HMW *β*-d-glucan included in a diet with phytosterols produced an additive effect on blood cholesterol reduction activity [118].

Natural polysaccharides can reduce the cholesterol level due to their ability to form a viscous medium in the intestine and thus capture bile acids. Mäkelä et al. [119] studied bile acid binding using an in vitro upper gastrointestinal tract model (Na_2_HPO_4_ buffer with adjusted pH 6.9; amylase, pepsin, pancreatin, and bile acid solutions to imitate digestion processes) and confirmed that the *M*_w_ of *β*-d-glucan and its concentration in food influenced the mobility of bile acids. Enzymatically degraded *β*-d-glucan had little influence on bile acid binding, while non-degraded and more viscous *β*-d-glucan caused the physical non-covalent binding of bile acids. According to Marasca et al. [106], neither acid hydrolysis nor the oxidative degradation of oat or barley *β*-d-glucans could improve bile acid retention, and this property is related to the viscosity of these polysaccharides. Mäkelä et al. [120] suggested that, consuming *β*-d-glucan in soluble form, the viscous consistency of the inoculum will limit the mobility of bile acids. However, if the food matrix contains this polysaccharide in an insoluble form, the retention of bile acids will reflect on its ability to form a gel. Hydrolysates of oat *β*-d-glucans (*M*_w_ 1450, 730, and 370 kDa) supplemented with a high-fat meal significantly reduced the body weight of mice [89]. According to further experiments, both hydrolysates and native *β*-d-glucan decreased serum LDL and VLDL levels and further improved the liver lipid profile in mice [121]. Hydrolysates increased fecal cholesterol and TGA excretion and thus improved the lipid profile more effectively than native *β*-d-glucan. The meta-analysis by Yu et al. [122], which included 13 trials with 927 participants, concluded that the consumption of oat *β*-d-glucan significantly reduces total cholesterol and LDL levels in adults with hypercholesterolemia without significant changes in TAG and HDL levels..

Hakkola et al. [123] studied the effects of *β*-d-glucans (*M*_w_ > 1000 kDa, 524 kDa, and 82 kDa) of oat bran concentrates in food on the fecal contents of bile acids CDCA, DCA, and CA in humans. The consumption of a meal with HMW *β*-d-glucan resulted in the highest excretion of bile acids and pressure in the duodenum, while the concentration of phenolic compounds in the urine was the lowest. The LMW *β*-d-glucan diet produced the opposite effect and the lowest DCA and CDCA amounts. The addition of MMW *β*-d-glucan to the diet had similar effects as HMW *β*-d-glucan, but the pressure in the duodenum appeared to be closer to the values measured in the cases of LMW and HMW *β*-d-glucan-containing meals. Rosa-Sibakov et al. [124] showed that oat bran concentrates containing *β*-d-glucans (*M*_w_ 400–500 kDa) still demonstrated a beneficial capacity of bile acid binding similar to oat HMW *β*-d-glucans (*M*_w_ ≥ 1000 kDa) but did not produce discomfort in the intestine due to a moderate colon enzymatic activity. Marasca et al. [112] prepared oxidized derivatives of barley and oat *β*-d-glucans using TEMPO or sodium periodate but showed that oxidation did not influence the binding capacity of *β*-d-glucan, and it was solely viscosity-dependent. The authors supported the idea that there is no effect of direct binding of bile acids by *β*-d-glucans.

Shen et al. [125] extracted two fractions of *β*-d-glucan from oat bran, water-soluble and water-insoluble. The authors did not measure the *M*_w_ of these fractions, but obviously water-insoluble oat *β*-d-glucan tends to have a higher *M*_w_. A study in mice showed that compared to mice with 1,2-dimethylhydrazine-induced colon carcinogenesis, all mice fed with oat *β*-d-glucans, soluble and insoluble, had significantly lower levels of fecal bile acid content. Meanwhile, the SCFA content was higher than in the model, especially for higher concentrations of soluble and insoluble *β*-d-glucan. Analyzing the incidence of colonic neoplasms in animal models, the authors found that compared to untreated (control) animals, mice fed with oat *β*-d-glucan in their diet had a lower incidence of tumors on average—up to a 66% reduction for high *β*-d-glucan dosage groups and a 33% reduction for low dosage groups. These findings could support the higher recommended amount of *β*-d-glucans in the human diet.

Oat *β*-d-glucan exhibits high bile acid-binding capability with the highest effect for HMW fractions, which makes flaked oats a good option for people with elevated cholesterol in the blood. Modifying oat processing to get an MMW-enriched product can thus reduce the discomfort in the duodenum caused by increased pressure. Low Molecular Weight *β*-d-glucans could show fewer benefits for cholesterol regulation. It raises questions about the need for consumer awareness about highly processed oat products and the reduction of *β*-d-glucan *M*_w_ with more processing steps and enzymatic activity of yeasts in bakeries.

## 9. Anti-Diabetic Activities

Among proven positive effects, *β*-d-glucans are potent in glycemic level reduction in the blood, thus in tandem with cholesterol regulation consistently reducing the possibility of developing cardiovascular diseases [126,127,128]. Regand et al. [129] studied food preparations containing different ratios of oat *β*-d-glucans of various *M*_w_ and starch to see how much this addition influences starch digestibility and postprandial glycemic response (PPGR). According to their results, the *M*_w_ of oat *β*-d-glucan correlates with starch digestibility in foods containing both these polysaccharides. The more viscous the *β*-d-glucan gel, the less resistant the starch is to digestion.

Based on the literature analysis, Noronha et al. [130] concluded that the *M*_w_ of oat *β*-d-glucans plays a decisive role in the effectiveness of reducing glycemic response. The minimal amounts of oat *β*-d-glucan needed for this purpose are 0.2 g, 2.2 g, and 3.2 g per 30 g of available carbohydrates (avCHO) for HMW (*M*_w_ > 1000 kDa), MMW (*M*_w_ 300–1000 kDa) and LMW (*M*_w_ 300 kDa) polysaccharides, respectively. Perez-Quirce et al. [55] reported that free glucose decreased in rice bread enriched with oat LMW *β*-d-glucans, and the starch digestibility index decreased in bread fortified with MMW and HMW oat *β*-d-glucans.

There is evidence that the 6 g/day dosage is much more effective in reducing glycemia and glycosylated hemoglobin than the 4 g/day recommended by the European Food Safety Authority [131]. It can be explained by the prolongation of gastric emptying, although there are still few articles with data about the long-term effects on other cardiovascular disease markers such as insulin sensitivity [132]. Zaremba et al. [133] found that even 4 g of HMW oat *β*-d-glucan added to food is effective in lowering appetite, compared to control food with the same amount of available carbohydrates per meal, i.e., 39.7 g in the control portion and 39 in that with *β*-d-glucan, with no effect on ad libitum eating. Therefore, foods containing HMW oat *β*-d-glucans can improve digestion and satiety, but in this study the impact of LMW oat *β*-d-glucan did not compare to that of HMW oat *β*-d-glucan.

Wolever et al. [134] confirmed the influence of oat *β*-d-glucans of various *M*_w_ on PPGR; they found that a decrease in *M*_w_ correlates with the time reduction for blood glucose to reach its peak, while HMW *β*-d-glucan demonstrated the opposite effect. Zurbau et al. [135], analyzing data from 103 trials (*N* = 538), found that oat *β*-d-glucans of different *M*_w_ can affect important diabetes biomarkers such as postprandial blood glucose and insulin response dramatically. The glucose incremental area under the curve (iAUC), insulin iAUC, and glucose and insulin incremental peak-rise (iPeak) values were significantly reduced (glucose iAUC and iPeak by 23% and 28%, and insulin by 22% and 24%, respectively). The authors noted that the *β*-d-glucan-containing diet viscosity does not always have an impact on glycemic peaks because the concentration of this polysaccharide is changed by its interaction with gastric fluid secretions. Nevertheless, the LMW oat *β*-d-glucan did not have an impact on iAUC and iPeak, while MMW (*M*_w_ 300–1000 kDa) and HMW (*M*_w_ > 1000 kDa) oat *β*-d-glucans were capable of regulating glucose and insulin levels in the blood.

Zhang et al. [136] modeled glucose diffusion and absorption in the rat small intestine by different concentrations of oat *β*-d-glucans of various *M*_w_ and found that glucose absorption reduced with the decreasing *M*_w_ of added polysaccharides. High Molecular Weight oat *β*-d-glucans reduced the concentration of available glucose in the small intestine more effectively than LMW oat *β*-d-glucans. Oat *β*-d-glucans inhibit starch digestion with a more pronounced effect of the HMW oat *β*-d-glucan. In the presence of oat *β*-d-glucans, the activity of Na^+^/K^+^ -exchanging ATPase (EC 7.2.2.13) reduced in the small intestine mucosa of rats, and the inhibition of this activity was more pronounced when its *M*_w_ and concentration increased. However, when the concentration of the *β*-d-glucan of defined *M*_w_ increased, the Na^+^/K^+^ ATPase activity increased because of higher gastrointestinal motility. The addition of oat *β*-d-glucans also reduced the activities of sucrose α-glucosidase (EC 3.2.1.48) and maltose α-glucosidase (EC 3.2.1.20), while the effect of HMW *β*-d-glucans was more pronounced than that of LMW *β*-d-glucans.

Overall, many studies are proving the *M*_w_-dependent lowering effect of oat *β*-d-glucans on the glycemic level, and more are expected in the future [137]. Based on the data available, HMW *β*-d-glucan appears to be the most active fraction. We can conclude that the diet should favor fewer processed oats in food due to the better retention of HMW *β*-d-glucan, which is much more effective in reducing glycemic response than LMW *β*-d-glucan [130].

## 10. Antioxidant Activity

The antioxidant activity of oat *β*-d-glucans with different *M*_w_ was tested by in vitro and in vivo experiments. Oat milling fractions, bran concentrate, and oat *β*-d-glucan extract contain a significant amount of polyphenols that may significantly impact the antioxidant activity of these preparations [104,105]. The antioxidant activity of oat *β*-d-glucans can be improved by complexation or mixing with tea polyphenols [102]; in vitro, the complex showed higher O_2_^•−^ scavenging activity, while the mixture was better in the scavenging of OH^•^ in vitro. In the in vivo experiments, the complex more effectively activates SOD (EC 1.15.1.1) and GPx (EC 1.11.1.9) in the liver than the mixture. However, the effects of both preparations on GPx activity, MDA level, and TAC in serum were comparable.

Du et al. [138] found that the results of the oxygen radical absorbance capacity (ORAC) and ferric reducing antioxidant power (FRAP) assays of fungal and oat *β*-d-glucans of various *M*_w_ strongly depend on the source of these polysaccharides, but the effect of *M*_w_ was less pronounced. However, oat HMW *β*-d-glucan (*M*_w_ 1800 kDa) exhibited the highest value of ORAC, while for MMW *β*-d-glucan (*M*_w_ 500 kDa) this value grew in a dose-dependent manner.

The effect of oat *β*-d-glucans as antioxidants was also evaluated in vivo on rats with LPS-induced chronic enteritis [139,140]. Błaszczyk et al. [139] studied the influence of LMW and HMW oat *β*-d-glucans on oxidative stress and antioxidant defense in the spleen. The spleen homogenates of experimental animals contained more LOOH, the non-stable lipid peroxidation products capable of cell membrane disruption, than control animals. In this study, the LMW fraction of oat *β*-d-glucan reduced LOOH levels, while the HMW fraction was more effective for LOOH regulation in healthy animals. The authors presumed that the mechanism behind the *β*-d-glucan scavenging activity was through the atom transfer reaction involving anomeric hydrogens in a polysaccharide macromolecule, which act as free radical quenchers. In a similar experiment, Suchecka et al. [140] studied how LMW and HMW oat *β*-d-glucans can influence the antioxidant activity of the stomach and liver tissues. High Molecular Weight *β*-d-glucans reduced the concentration of lipid peroxidases in the stomach but not in the liver of the *β*-d-glucan-treated animals compared to the control group. Instead, LMW *β*-d-glucans decreased levels of lipid peroxidation products, i.e., LOOH and TBA, in both the stomach and liver compared to the control group. Diets with LMW and HMW oat *β*-d-glucans can decrease the toxic effect of LPS on the liver of rats with enteritis and reduce the concentrations of 7-ketocholesterol and 25-hydroxycholesterol by improving the process of cholesterol auto-oxidation.

The hepatoprotective and antioxidant properties of oat *β*-d-glucan may support the idea of the benefits of oat consumption in preventing the damaging effects of toxic environments and food, and its antioxidant activity at the tissue level may prolong human life.

## 11. Prebiotic Activity

The increasing interest in the microbiome and its composition has raised the issue of sustaining a healthy intestinal microflora supported by appropriate prebiotics, e.g., fibers or inulin. Generally, oat *β*-d-glucan can stimulate the growth of “good” human gut microbiota, e.g., *Lactobacillus* and *Bifidobacterium*. Many studies have focused on oat bran and isolated *β*-d-glucan prebiotic activity, but only a few touch on the role of the *M*_w_ in this phenomenon [141,142,143].

Oat *β*-d-glucan can influence the cardiovascular system via microbiota in a way that suggests it is a cardio-protector [144]. It can be fermented by intestinal bacteria in the colon, increasing the concentration of SCFA, i.e., acetate, butyrate, and propionate. These molecules are involved in the regulation of glucose and cholesterol metabolism via free fatty acid receptors FFA2 and FFA3 [145]. Activation of FFA2 can enhance insulin secretion and inhibit ghrelin secretion. Ligand-activated FFA2 downregulates NFkB by recruiting *β*-arrestin 2, leading to a reduction of pro-inflammatory cytokine expression. FFA3 in pancreas islets, when activated by propionate, inhibits insulin secretion. Dong et al. [146] revealed that oat *β*-d-glucan hydrolysates are better substrates (even as a single carbon source) for probiotic bacteria strains than the native oat *β*-d-glucan. Immerstrand et al. [90] studied the effect of a series of oat bran preparations characterized by *β*-d-glucans of *M*_w_ 2348, 1311, 241, 56, 21, and <10 kDa on plasma lipids and the fecal formation of SCFA in C57BL/6NCrl mice. They concluded that diets with oat *β*-d-glucan with *M*_w_ from 1311 kDa down to 10–20 kDa were equally effective in reducing plasma cholesterol in mice that developed high blood cholesterol due to an atherogenic diet. Despite that, the ratio of propionic acid and butyric acid contents to the acetic acid content increased with increasing *M*_w_ of *β*-d-glucans, leading to the conclusion that as a component of a usual Western diet, the higher *M*_w_ of oat *β*-d-glucan is more beneficial for microbiota. Wilczak et al. [113] have shown that both LMW and HMW oat *β*-d-glucans increased the numbers of LAB in feces in healthy rats and rats with enteritis. The administration of *β*-d-glucans improved the profile of SCFA in rat feces, characterized by decreasing hydroxybutyric acid levels and increasing lactic and propionic acid content. Increasing propionic acid can be explained by improved fermentation.

Lazaridou et al. [147] studied the influence of oat *β*-d-glucans in a fermented product on the viability of probiotic and yogurt-starter bacteria strains. According to their results, the addition of *β*-d-glucan (*M*_w_ 117 kDa) slightly reduced the number of living cells in the *S. thermophilus* population, did not affect *Lactobacillus bulgaricus* viability, and, interestingly, had a positive effect on probiotic strain *Lactobacillus paracasei* growth. Bai et al. [148] found that oat *β*-d-glucan mostly induced the elevation of the butyrate level but did not affect acetate and propionate levels in mice intestines compared to a blank control. In the human gut, *β*-d-glucan fermentation elevated the propionate level compared to the untreated control group. The authors measured the degradation of *β*-d-glucans in the intestines of humans and mice at different time points. Fecal microbiota can degrade *β*-d-glucans in humans faster than in mice, i.e., after 3 h of fermentation the concentration of *β*-d-glucans decreased to 3.29 mg/mL and 1.39 mg/mL in the mouse and human groups, respectively, from the initial 10 mg/mL. After 24 h, human microbiota completely degraded oat *β*-d-glucan, but some traces of this polysaccharide were still detectable in the mouse group, suggesting that human microbiota is more adapted for the complete reduction of oat *β*-d-glucan. It is important to mention the results of Akkerman et al. [149], which showed that human infants of 2 and 8 weeks old possess microbiota that are incapable of degrading native oat *β*-d-glucan (*M*_w_ ≥ 300 kDa). The bacterium *Clostridium histolyticum*, which uptakes *β*-d-glucan in adults, is absent in the infant gut microbiome. Instead, when oat *β*-d-glucan was treated with β-glucanase to obtain fractions of *M*_w_ < 300 kDa, the complete degradation of this polysaccharide occurred even for the fecal microbiota of infants. Changes in microbiota composition in infants were mainly due to the population increase in bacteria from the genus *Enterococcus*, which is related to immunomodulatory functions. Dong et al. [146] found that after microwave processing and the reduction of *M*_w_, oat *β*-d-glucan becomes eutrophic for butyrate-producing bacteria *Blautia faecis* sp. and *Dialister* spp. The fermentation of LMW oat *β*-d-glucan slurries resulted in a higher volume of SCFA than that of HMW oat *β*-d-glucan slurries. The authors concluded that the effect of thermal processing, also used in their study, did not markedly reduce the prebiotic potential of the oat *β*-d-glucan, but microwave processing can only enhance its positive effect.

Adding oat *β*-d-glucan to the diet of dairy calves increased serum levels of total protein, albumin, globulin, SOD, and diamine oxidase (EC 1.4.3.22), with changes in gut microbiota abundance and plasma metabolic profile correlated with increases in these markers [150]. Oat *β*-d-glucan plays a beneficial role in the interaction between the gut microbe and the host and thus may serve as a prebiotic in dairy calf feeding.

Several investigations evaluated the prebiotic potential of oat fiber and found that many normal bacteria ferment these polysaccharides and increase SCFA concentration, while there is evidence that fragments of hydrolyzed polysaccharides are better substrates for such bacteria. Therefore, foods containing hydrolyzed oats improve gut microbiota, whereas oat products with HMW *β*-d-glucan are suitable for cholesterol regulation.

## 12. Anticancer Activity

*β*-d-Glucans demonstrated suppressive effects on tumor growth and even the reduction of primary tumor mass, though this activity can differ depending on the source of *β*-d-glucans [151]. For example, Shah et al. [152] proved oat *β*-d-glucan to be a more potent anti-proliferative agent on the cell line Colo-205 than barley *β*-d-glucan.

Despite reports about *β*-d-glucan being effective in combination with anticancer treatments, only a few clinical trials to consistently study the anticancer activity of *β*-d-glucan were established [153]. Unfortunately, despite the authors often underlining the importance of the *M*_w_ of the used *β*-d-glucan fraction, few works show the comparison in the activity of oat *β*-d-glucans with different *M*_w_ in the range of the same study.

Parzonko et al. [154] studied oat LMW *β*-d-glucan (*M*_w_ 134 kDa) as an anticancer agent on human HTB140 melanoma cells using an MTT assay. At the *β*-d-glucan concentration of 200 μg/mL, the viability of cells was 50%, and the cytotoxic effect of this polysaccharide was concentration-dependent, as well as lactate dehydrogenase (EC 1.1.1.27) release into the culture media. There were also changes in the mitochondrial functions of HTB-140 cells. Intracellular ATP levels negatively correlated with the rise of the concentration of *β*-d-glucan, with the lowest value 50.8 ± 7.8% at 200 μg/mL. The authors stated that the same experiment conducted in parallel on the cell line of normal fibroblasts had shown no cytotoxicity. Choromanska et al. [67] studied the anticancer activity of the LMW oat *β*-d-glucan, analyzing its possible toxicity for four cell lines: human pigmented malignant melanoma Me45, human epidermoid carcinoma A431, normal human keratinocyte HaCaT, and normal mouse macrophage P388/D1. The results suggested that oat *β*-d-glucans decreased the viability of both cancer cell lines and did not show a toxic effect on normal cells. In another study, Choromanska et al. [155] evaluated how LMW and HMW oat *β*-d-glucans affect normal keratinocytes, two lung cancer cell lines, and red blood cell hemolysis. The authors also confirmed tolerance to oat *β*-d-glucan by normal cells. Red blood cell hemolysis tended to be slowed to 51.64% by *β*-d-glucan treatment, with HMW being more effective in high concentrations than LMW *β*-d-glucan. In addition, MDA, the final product of lipid peroxidation, increased in a dose-dependent mode administrating HMW *β*-d-glucans to H69AR cells. Low and high concentrations of LMW *β*-d-glucans caused nucleus perturbations and abnormal chromatin condensation in A549 and HaCaT cells. All concentrations of HMW fractions caused actin fiber abnormalities in H69AR and HaCaT cells. A study on Me45 cells has shown that LMW oat *β*-d-glucans at a high concentration of 200 µg/mL in cell-growth media decreased the viability of melanoma cells to 19%, while in combination with electroporation to 12.5%, and caspase 3 expression increased significantly. At the concentration of 100 µg/mL, half of all cells were stained positively for cytochrome c. For caspase 12, a positive reaction was evident at 100 µg/mL and 200 µg/mL of oat *β*-d-glucans in media. This work evidences that LMW oat *β*-d-glucans have the potential to initiate the apoptotic process in melanoma cells involving cytochrome c, which participates in caspase activation, followed by the activation of dormant killer proteases [156].

Zhang et al. [157] evaluated the anticarcinogenic effect of three oat *β*-d-glucans with *M*_w_ of 10 kDa, 200 kDa, and 500 kDa on mice inoculated in the flanks with B16F10 tumor cells. The polysaccharide solutions were injected intra-peritoneally in the tumor-bearing animals at 25 mg/kg. The control group of mice received PBS or dextran solution. A part of the mice were intravenously injected in the tail with B16F10 cells to generate lung metastasis. Similarly, in an osteosarcoma metastasis model, 5 K7 M2-luc2 mouse osteosarcoma cells were intravenously injected into Balb/c mice; the animals received 25 mg/kg of oat *β*-d-glucan (*M*_w_ 200 kDa) intranasally. This preparation inhibited tumor growth the most effectively. Mice injected with B16F10 melanoma intravenously after treatment with the 200 kDa *β*-d-glucan demonstrated the lowest incidence of lung metastases. Tumors treated with oat *β*-d-glucans also showed microenvironmental differences, particularly regarding pro-inflammatory factors IFN-γ, TNF-α, and Th-1 chemokines (CXCL9 and CXCL10). The IRF1 and PDL-1 levels increased, and T cells, which produced Granzyme B and IFN-γ, infiltrated tumors. CD11 b+ and CD11 c+ cells, DC, T cells, and presumably NK cells supported the immune control of B16F10 treated with oat *β*-d-glucans. The authors hypothesized about an unidentified signaling pathway involved in the activation of DC by *β*-d-glucan-activated macrophages since conditioned media from later were more potent in DC activation than DC directly treated with *β*-d-glucan.

It is hard to speculate about the effectiveness of oat *β*-d-glucan of specific ranges of *M*_w_ for cancer treatment. The mechanism of cytotoxicity on cancer cells can be related not only to the activation of immune cells by *β*-d-glucan molecules but also directly to GLUT1-related transport inside the cell. Since oat *β*-d-glucan shows no cytotoxicity on normal cells, further study on anticarcinogenic activity needs to investigate the specific effects of the various *M*_w_ of *β*-d-glucan to address their use. For example, clarifying the cytotoxic effect through GLUT1 transporter involving different *M*_w_ of oat *β*-d-glucan could be a beneficial contribution.

## 13. The Application Prospect of Food Technology

Since oat *β*-d-glucan, pure or in oat-enriched flour, has become available for the study and development of food technologies, it is interesting to trace the growing interest in this polysaccharide. If not tailored with strict specificity in mind [158], oat β-d-glucan formulations have been customized for a broad range of needs, relevant in oat-containing beverages [159,160,161,162], bakery products [163,164,165], and breakfast cereals [166]. There was a whole line of products and ingredients based on oat fibers, including the Nutrim product line from several producers, the former C-Trim^TM^, and Oatrim^TM^ (Betatrim^TM^), each with variations of *β*-d-glucan concentration and properties related to processing conditions. For example, not very well-known outside the U.S., a fat replacer based on oat flour with the commercial name Oatrim^TM^ (currently unavailable) was a mixture of oat maltodextrins and *β*-d-glucan, obtained by the enzymatic treatment of oat flour or bran with α-amylase. Due to its advertised properties, such as being structurally similar to common fats used in bakery and conditioner branches, it was possible to add it to the widest variety of foods: spreads, creams, yogurts, sauces, cakes, and meat products. This product contains 1–10% oat *β*-d-glucan and has proven to have similar health benefits to other products enriched with this polysaccharide [167]. It is necessary to mention that products named Oatrim^TM^ or Betatrim^TM^ are currently impossible to find on the pages of the company Quacker^TM^, possibly due to problems with FDA approval up to 2003 and later discontinuation. Nutrim-OB^TM^, another product prepared by the complex thermal treatment of oat mass, contains *β*-d-glucan with reduced *M*_w_ and possesses several times higher viscosity than unprocessed oats and enhanced bioactivity [168]. The C-Trim^TM^ product line offers the highest concentration of oat *β*-d-glucan, up to 50% as advertised (up to 60% was found by Kim et al. [167] in the C-Trim^TM^ 3C2) due to more effective starch separation. Such products, to some extent, tend to have reduced oat *β*-d-glucan *M*_w_ with the increasing number of processing steps and increasing of *β*-d-glucan concentration, from 800 kDa in Oatrim^TM^ to 240 kDa in C-Trim^TM^ 3D1 preparation, though currently on the pages of FutureCeuticals Inc. there is no mention of the C-Trim^TM^ product line and only Nutrim^TM^ 5 and Nutrim^TM^ 10 are found in the product guide. A product with a similar name, Nutrim^TM^ 30 (30 is the number of servings per package, where one serving is 7.5 g), from Nutrim^TM^ LLC advertises up to 750 mg of oat *β*-d-glucan per serving, which is 10% of total weight. Also, Van Drunen Farms (Momence, IL, USA) has Nutrim^TM^ Oat powder products with numbers 20 and 40, sold as ingredients and listed on the ShelfLife.us marketplace. Unfortunately, there are no other specifications of this ingredient on the web, such as the percentage of oat fiber in powders or the *M*_w_ of *β*-d-glucans, and there is not even a mention in recent articles, indicating a decrease in research activity in this area compared to the period from 1990 to 2010. Overall, this tendency in decreasing studies signals a corresponding fall of interest from potential investors, which halts progress in creating a higher range of intelligently developed oat foods.

## 14. Conclusions

In the last decade, interest in studying the biological activities of mixed-linkage oat *β*-d-glucans has become more consistent. The findings reported by the recent literature support the idea that the *M*_w_ of *β*-d-glucan indeed correlates not only with fundamental properties of food containing oats, such as the feeling of satiety after eating or the glycemic index induced by food, but also the fermentation activity in the intestines and the influence on intestinal microbiota maintenance. The *M*_w_ of *β*-d-glucan directly influences the antioxidant effects in the liver and stomach tissues, the rate of bile acid formation, and the blood cholesterol level. Understanding the properties of bioactive oat *β*-d-glucans and standardizing preparation and possible modifications of these polysaccharides is essential for their appropriate use in future foods and non-food oat preparations, considering that oats are the most available cereal source of mixed-linkage *β*-d-glucans.

Despite this, a very relevant problem arose. While 10–20 years before, there were studies of a variety of oat-based food ingredients, the hype raised from advances in oat processing and the possibility of oat ingredients substituting fats and gums in a plethora of food types (including liquids and emulsions) quickly and drastically declined. Currently, rising interest in oat *β*-d-glucan for food applications has led to improved oat-processing technology. Now there are many ingredients enriched with this polysaccharide, but, unfortunately, only viscosity and percentage of fiber, not *M*_w_, are mentioned in such products. Due to the increasing amount of data supporting biological activities for LMW and HMW oat *β*-d-glucans, food packaging should indicate *β*-d-glucan polysaccharide categories according to their *M*_w_.

A perspective for the use of oat *β*-d-glucan containing products lies in the fact that a wide range of such foods already exists, and the improvement in the last few years is after the increased popularization of *β*-d-glucan for its proven health benefits. In our opinion, the next logical step should be further informing consumers about the health benefits of oats and that they are the cheapest source of *β*-d-glucan on the market. People with a risk of gut disease or generally suffering from digestion problems, overeating, or high cholesterol could select oat-containing food or drinks according to the *β*-d-glucan parameters most suitable for their particular situation. For example, LMW *β*-d-glucan-enriched food will be more effective on the molecular level in cases of inflammation in the gut, while HMW *β*-d-glucan addition to regularly consumed food can help to regulate the cholesterol level in the blood. A not secondary issue is the stabilization of the regular gut microflora by these products due to their prebiotic properties. From all the data discussed above, it appears that *β*-d-glucans from oats are preparations that, if well classified, have an excellent prospect for being used as beneficial supplements in specific medical treatments.

## Figures and Tables

**Table 1:** Overview of *β*-d-glucans of various structure and origin.

Structure	Product	Origin	*M*_w_ (kDa)	References
(1 → 3)-*β*-d-Glucans	Curdlan	*Agrobacterium tumefaciens*	2000	[14]
	Paramylon	*Euglena gracilis*	500	[15]
	Pachyman	*Poria cocos*	89–168	[16]
(1 → 4)-*β*-d-Glucans	Plant cellulose	Higher plants, seaweeds		[8]
	Animal cellulose	Tunicata		[10]
	Microbial cellulose	*Acetobacter xylinum*		[9]
		*Sarcina ventriculi*		
(1 → 6)-*β*-d-Glucans	Pustulan	*Lasallia pustulata*		[17]
	Lasiodiplodan	*Lasiodiplodia theobromae*		[18]
Mixed-linkage *β*-d-glucans	Lichenan	*Cetraria islandica*		[7]
	Cereal *β*-d-glucans	*Hordeum vulgare*	1700–2700	[11]
		*Avena sativa*	65–3100	[12]
		*Secale cereale*	800–1300	[12]
		*Triticum* sp.	209–487	[13]
Branched *β*-d-glucans	Laminarin	*Laminaria digitate* *Saccharina longicruris* *Durvillaea antarctica*	3–7	[5,6]
	Calocyban	*Calocybe indica*	200	[19]
	Botryosphaeran	*Botryosphaeria* sp.		[20]
	Epiglucan	*Epicoccum nigrum*		[21]
	Grifolan	*Grifola frondosa*		[22]
	Krestin	*Coriolus versicolor*	10–100	[23]
	Lentinan	*Lentinus edodes*	300–800	[24]
	Pestalotan	*Pestalotia* sp. 815		[25]
	Pleuran	*Pleurotus ostreatus*	600–700	[26]
	Schizophyllan	*Schizophyllum commune*	450	[27]
	Scleroglucan	*Sclerotium glucanicum*		[28]
	Zymosan	*Saccharomices cerevisiae*	240	[29]

## Data Availability

Data available upon request to corresponding authors.

## Supplementary Materials

The following supporting information can be downloaded at: https://www.mdpi.com/article/10.3390/foods12061121/s1, Table S1: Comparison of biological activities of oat *β*-d-glucans in relation to *M*_w_.

## Abbreviations

5 K7M2-luc2, murine osteosarcoma cells; A431, human epidermoid carcinoma cells; A549 adenocarcinomic human alveolar basal epithelial cells; Aβ, amyloid β; ACE, angiotensin-converting enzyme; ATPase, Adenosine 5′-triphosphatase; avCHO, Available carbohydrates; B16F10, tumor cell line; BALB/c, laboratory-bred albino strain of the house mouse; C57BL/6NCrl, aged laboratory-bred strain of the house mouse; CA, cholic acid; CD11 the α component of integrins mediating leukocyte adhesion; CDCA, chenodeoxycholic acid; CM, carboxymethyl; Colo-205, lymphoid cell line from a patient with colon carcinoma; CR3, immunological cell surface receptor for a complement component; GLUT1, glucose transporter 1; CXCL, chemokine (C-X-C motif) ligand; DC, dendritic cell; DCA, deoxycholic acid; Dectin-1, C-type lectin receptor; DS, degree of substitution; FFA2, FFA3, free fatty acid receptors 2 and 3; FRAP, ferric ion reducing antioxidant power; *G*′, storage modulus; *G*″, loss modulus; GFAP, glial fibrillar acidic protein; GPx, glutathione peroxidase; HaCaT, cell line of human normal keratinocytes; HDL, high-density lipoprotein; HMW, high molecular weight; H69AR, epithelial cells line originated from the lung of a carcinoma patient; HCT-1116, human colorectal carcinoma cell line; iAUC, glucose incremental area under curve; IFN, interferon; IL, interleukin; IRF1, interferon regulatory factor 1; iPeak, insulin incremental peak-rise; kGy, kilogray; LC3B, microtubule-associated protein 1 light chain 3, mammalian isoform B; LAB, lactic acid bacteria; LDL, low-density lipoprotein; LDLR, low-density lipoprotein receptor; LMW, low molecular weight; LOOH, lipid hydroperoxides; LPS, lipopolysacharide; MAPK, mitogen-activated protein kinase; MCF7, epithelial cell line isolated from the breast tissue of a patient with metastatic adenocarcinoma; MDA, malondialdehyde; Me45, human pigmented malignant melanoma cell line; MMW, medium molecular weight; MTT, 3-[4,5-dimethylthiazol-2-yl]-2,5 diphenyl tetrazolium bromide assay; *M*_w_, molecular weight; MYD88, myeloid differentiation primary response 88; NF-κB, nuclear factor κ-light-chain-enhancer of activated B cells; *Nlrp1*, nucleotide-binding domain and Leu-rich repeat (NLR) containing protein 1 (gene); NK, natural killer cell; ORAC, oxygen radical absorbance capacity; P388/D1, mouse macrophage cell line; p38, a kind of MAPK; PBS, phosphate-buffered saline; PDL-1, programmed death ligand 1; PI3K-Akt, phosphoinositide 3-kinase (PI3K) and protein kinase B (Akt) activation pathway; PPGR, post-prandial glucose response; PRR, pattern recognition receptor; RAF, rapidly accelerated fibrosarcoma; SCFA, short chain fatty acid; *Serp2*, stress-associated endoplasmic reticulum protein 2 (gene); SOD, superoxide dismutase; Syk-CARD9, spleen tyrosine kinase (SYK) and caspase recruitment domain-containing protein 9 (CARD9) mediating pathway; TAC, total antioxidant capacity; TAG, triacylglycerols; TBA, thiobarbituric acid; TEMPO, 2,2,6,6-tetramethyl- piperidine-1-oxyl; Th-1, type 1 thymus helper cells; TLR, Toll-like receptor; TNBSA, 2,4,6-trinitrobenzenesulfonic acid; TNF-α, tumor necrosis factor α; WZB117, 3-fluoro-1,2-phenylene bis(3-hydroxybenzoate); VLDL, very-low-density lipoprotein.
