# Supplementary material for: Perspectives in the Application of High, Medium, and Low Molecular Weight Oat β-d-Glucans in Dietary Nutrition and Food Technology—A Short Overview
Source: Foods. 2023 Mar 7;12(6):1121. doi: 10.3390/foods12061121 (PMC10048208; doi:10.3390/foods12061121)
Supplement: Supplementary file 1 [file foods-12-01121-s001.zip › foods-2231966-supplementary.pdf]

Supplementary materials

Table S1. Comparison of biological activities of oat β-D-glucans in relation to M_w.

MW range (kDa)			Source/samples	Isolation/treatment	Administration/assay	Results	Ref.
**LMW**	**MMW**	**HMW**					
70	100, 133, 199		Milled oat	Extraction with water, γ-ray irradiation	In vitro assays	• Antioxidant activity increases with γ-radiation intensity	96
80, 61, 52	108, 144, 200			Alkaline extraction, γ-ray irradiation		• Proven biocompatibility with human keratinocytes HaCat<br>• Cytotoxic activity against cancer cell lines colo-205 and MCF7	97
83	192	650	Oat β-D-glucans	Including in rice breads	Starch digestibility in vitro, PBGR	• LMW β-D-glucan reduces free glucose in rice breads<br>• HMW β-D-glucan decreased starch digestibility	55
82	325	1996		Enriched dough, β-glucanase		• M_w of oat β-D-glucans negatively correlated with digestible starch<br>• Formulations with HMW β-D-glucan reduced PBGR effectively	129
	221-225	389-398	Instant oatmeal	Cooking with boiling water<br>Mixing with cold milk	Oral consumption	• Higher satiety of instant oatmeal than same caloric intake due to higher viscosity of β-D-glucans	133
59		1700	Plant matrices	Alkaline extraction, removal of proteins<br>Mixing with feed (1% w/w)		• LMW β-D-glucans showed benefits in recovery from colitis<br>• HMW β-D-glucans ameliorated inflammation in mucosa and submucosa	108
						• LMW β-D-glucans reduced pro-inflammatory cytokines<br>• HMW β-D-glucans enhanced tissue recovery	109
52, 76	153	393, 841<br>1980		Partial hydrolysis with β-glucanase<br>Mixing with water (preload)	Oral consumption<br>PBGR	• Oat β-D-glucans influence PBGR in healthy humans<br>• Lower M_w of β-D-glucans correlated with decreasing of time for blood glucose to peak after preload	134
	370	730, 1450	Oat β-D-glucans	Partial hydrolysis<br>Supplementation to high fat meal	Oral consumption by mice	• Hydrolysates reduced the body weight and improve lipid profile effectively than native β-D-glucan	89, 121
	Not specified			Mixing with animal feed (AIN-93M)		• LMW β-D-glucans improved appetite for colitis mice<br>• Both diets increased number of T cells in lymphocyte population, B cells and NK cells (higher in mice fed with LMW β-D-glucans)	110
						• Both β-D-glucans enhanced autophagy related genes expression and reduced of Caspase-3 expression and apoptosis in Crohn`s disease mice	111
				Partial hydrolysis with β-glucanase<br>Homogenized and diluted in water	*In vitro* models of bile acid binding	• Enzymatically degraded β-D-glucans slightly affected bile acids binding<br>• Viscous non-degraded β-D-glucans caused demobilization of bile acids	119
6	173, 275	1584	Oat flour	Extraction, acidic hydrolysis, oxidation		• Modifications of oat β-D-glucans did not improved bile acids binding<br>• Viscosity played significant role in this property	106
	158, 173	1180, 1840	Oat bran concentrate	Acidic and/or enzymatic hydrolysis		• All β-D-glucans, especially HMW, decreased bile acid mobility	120
82	524	>1000		Treatment with cell wall degrading enzymes	Oral consumption by humans<br>Exp. diet 3-4 days	• HMW β-D-glucans stimulated excretion of bile acids, increased pressure in duodenum and lowered concentration of phenolic comp. in urine<br>• LMW β-D-glucans lowered bile acids and pressure in duodenum, but enhanced excretion of phenolic acids in urine<br>• Effect of MMW β-D-glucan was similar to that of HMW β-D-glucan but with lower pressure in duodenum	123
<100	200-500	1000			In vitro models of bile acid binding/retention	• LMW (HMW) β-D-glucans had low (high) bile acid binding capacity<br>• MMW β-D-glucan showed detectable bile acid reduction, high intestinal viscosity and tolerable fermentation activity without physical discomfort	124
70					In vitro assays	• LMW β-D-glucan (50-100 µg/ml) increased viability of HaCaT cells	67

						• It showed cytotoxic effect on A431 and Me45 cell lines, but was safe and no toxic for normal cell lines	
		Not specified				• LMW and HMW *β*-D-glucans decreased cell viability in A549 and H69AR cell lines; no cytotoxic effect on HaCaT cells<br>• HMW *β*-D-glucan increased MDA for H69AR and for A549<br>• LMW *β*-D-glucan altered nucleus structure in A549<br>• HMW *β*-D-glucan caused abnormalities of cytoskeleton in H69AR	155
81, 191	500	1040, 1508, 1800	Oat *β*-D-glucans			• Oxygen radical uptake by oat *β*-D-glucans slightly correlated with M_W	138
68, 187	325, 461	5687	Oat bran	Extraction, acid hydrolysis<br>Mixing with food	In vitro model of glucose absorption and diffusion in rat small intestine	• HMW *β*-D-glucan decreased concentration of available glucose in small intestine; LMW *β*-D-glucan was less effective<br>• Digestion of starch proceeded slower in presence of both *β*-D-glucans<br>• Na^+/K^+ ATPase activity was dependent on M_W and concentration of *β*-D-glucans and decreased with increasing of both values<br>• Concentration of *β*-D-glucans with defined M_W positively correlated with Na+/K+ ATPase activity due to gastrointestinal motility<br>• *β*-D-Glucans especially HMW decreased activities of sucrase and maltase	136
70		2180	Oat fibers	Alkali extractions, freeze-milling<br>Exp. diet with 1% oat *β*-D-glucan	Oral consumption by rats/mice	• HMW *β*-D-glucans reduced inflammation in colon altering cytokines levels: elevating IL-10 and reducing IL-2 and TNF-*α*<br>• Diet supplementation with LMW and HMW oat *β*-D-glucan promoted significant increase in fecal LAB in healthy and enteritis rats	113
						• LMW *β*-D-glucans reduce lipid hydroperoxidases in rats spleens after LPS injections	139
						• HWM *β*-D-glucan decreased expression of Granzyme C-like protein reducing inflammation, reduced expression of gene *Serp2* (inhibition of immune response in mice with enteritis), and boosted expression of gene *Nlrp1* (inhibition of inflammation and tumorigenesis)<br>• LMW *β*-D-glucan increased IL34 and downregulated prostaglandin E receptor 3, both related to inflammatory processed.	114
						• HMW *β*-D-glucan decreased lipid peroxidases in stomach of enteritis rats<br>• LMW oat *β*-D-glucan lowered LOOH and TBA in stomach and liver<br>• Both HMW and LMW oak *β*-D-glucans decreased concentration of toxic 7-ketocholesterol and 25-hydroxicholesterol	140
						• HMW *β*-D-glucan reduced NK in blood of healthy rats<br>• Both *β*-D-glucans decreased total and B lymphocytes and granulocytes in enteritis rats with more pronounced effect for HMW *β*-D-glucan<br>• Monocytes decreased in number in rats fed with HMW *β*-D-glucan	115
						• LMW and HMW *β*-D-glucans reduced level of lipid oxidation in stomach, with more pronounced effect for HMW *β*-D-glucan	116
10, 200	500			Extraction with hot water<br>Labelling with FITC [109]	Solutions of oat *β*-D-glucans were injected intra-peritoneally<br>Control was PBS or dextran solution	• LMW *β*-D-glucan (M_W 200 kDa) effectively inhibited primary tumors growth; lowest incidence of lung metastases; IFN-*γ*, TNF-*α*, Th-1 chemokines: CXCL9 and CXCL10; IRF1, PDL-1 levels were increased, tumors were infiltrated with T cells, which produced Granzyme B and IFN-*γ*<br>• CD11 b+ cells, dendritic cells, CD11 c+, T cells and presumably NK cells supported immune control of B16F10 treated with oat *β*-D-glucan	157
	165	1713	Oat kernels	Enzymatic and hot water extractions<br>Microwave heating, steaming	Fermentation system with fecal microbiota	• Microwave treatment reduces M_W of *β*-D-glucan and promotes growth of butyrate-producing bacteria Blautia and Dialister	146

				• Fermentation slurries with MMW *β*-D-glucan yielded more SCFA	
<300	300-1000	>1000	Meta-analysis	• MMW and HMW oat *β*-D-glucans significantly reduced glucose iAUC, insulin iAUC and glucose and insulin iPeak values	122
